# Allisartan ameliorates vascular remodeling through regulation of voltage-gated potassium channels in hypertensive rats

**DOI:** 10.1186/s40360-021-00498-7

**Published:** 2021-06-09

**Authors:** Xiaoqin Zhang, Ziying Zhao, Chunfang Xu, Fengping Zhao, Zhiqiang Yan

**Affiliations:** 1 Department of Cardiology, Southern Medical University affiliated Fengxian Hospital, Shanghai, 201499 China; 2 Shanghai University of Medicine and Health Sciences Affiliated Sixth People’s Hospital South Campus, Nanfeng Road No.6600, Shanghai, 201499 China; 3 Endoscopy Center, East Hospital, Tongji University School of Medicine, Shanghai, 200120 China

**Keywords:** Allisartan, wire myography, Vascular remodeling, Hypertension, Potassium channels

## Abstract

**Background:**

The objective of the present study was to determine the effect of allisartan, a new angiotensin II type 1 receptor antagonist on vascular remodeling through voltage gated potassium channels (Kv7) in hypertensive rats.

**Methods:**

The study included a total of 47 Sprague Dawley (SD) rats. The animals were randomized to sham operation (*n* = 14), untreated hypertensive control group (*n* = 18) and allisartan treatment group (*n* = 15). Using renal artery stenosis, hypertension was induced in animals. Single dose of allisartan was administered intra-gastrically to animals in the allisartan treatment group and match placebo in the other 2 groups. Wire myography was used to measure the muscle tension in isolated mesenteric arteries from the animals. Real-time polymerase chain reaction was used to quantify the expression of Kv7 channel mRNA subunits.

**Results:**

After 4 weeks of treatment, a significant decrease in mean arterial, systolic and diastolic blood pressure (SBP and DBP) was observed in allisartan treatment group compared to hypertension control group. The median arterial wall thickness and area/diameter ratio reduced significantly in treatment group compared to untreated hypertension group (*P* < 0.05). Wire myography demonstrated increased relaxation of mesenteric artery with increase in concentration of ML213. A significant up-regulation in the expression of all Kv7 mRNA subunits was observed in allisartan group compared to untreated hypertension group.

**Conclusions:**

From the results, allisartan was found to lower BP and preserve vascular remodeling through Kv7 channels.

## Background

Hypertension, a cardiovascular disease, endangers public health and is a leading cause of death [1]. Epidemiological studies indicated hypertension as a preventable risk factor for cardiovascular disease (CVD) [2]. Further, in hypertension, adaptive responses to hemodynamic and non-hemodynamic stimuli leads to excessive remodeling of vascular endothelial cells and alters microcirculation which leads to impaired tissue perfusion (due to enhanced vascular tone and reduced vasodilator response) resulting in end organ damage [3]. Furthermore, reported evidence suggested that changes in vascular voltage gated potassium channel (Kv) expression and/or function may contribute to hypertension of vascular smooth muscle from rat mesenteric arteries (MA) [4, 5], rat thoracic aorta [6], mouse aortic arteries [7] and mouse MA [8]. Kv7 channels functions as a conductor of K^+^ as a response to membrane depolarization, thus causing hyperpolarization and stabilization of the membrane potential. Hence Kv7 plays a key role in regulating the excitability of cardiomyocytes, smooth muscle cells and neurons [9]. It is reported as a major determinant of vascular tone [10]. Thus, alterations in the expression of Kv7 channels may contribute to cardiovascular risk factors like hypertension [9].

Angiotensin II, a key effector of renin-angiotensin system (RAS) that binds to angiotensin II type 1 receptor, contributes to the development of hypertension and related cardiovascular disease. Its action is mediated through AT1 receptors that are widely expressed in the kidneys especially in the smooth muscle cells of the arterioles [11]. It stimulates cellular hypertrophy [12], protein synthesis, activation of NADPH oxidase system to generate ROS [13] and synthesize collagen in the vascular smooth muscle cells [14]. Reduction of cardiovasular events was reported with blockade of the RAS [15, 16]. Several antihypertensive agents including angiotensin II receptor blockers (ARBs) and angiotensin-converting enzyme (ACE) inhibitors acts by blocking the RAS and are widely used in the management of hypertension [17]. ARBs improve both microvascular and macrovascular outcomes in hypertensive patients [18]. Studies also reported ARBs to be superior in ameliorating endothelial function and vascular damage [19–21]. Despite of their wide usage as first-line therapy, the cardiovascular protective effects of these agents still remained elusive [22].

Losartan, an orally active ARB is widely used due to its well-established efficacy and safety profile in hypertensive patients [15, 23]. In humans, cytochrome P450 (CYP450) metabolizes losartan to various metabolites along with an active carboxylic acid metabolite, EXP3174 that includes around 14 % of losartan [24]. EXP3174 is an selective angiotensin II type 1 receptor antagonist and was reported to be more potent than losartan both *in vitro* (15 times) and *in vivo* (30 times) [15, 25].

Unlike other ARBs, allisartan has advantage of low incidence of drug interactions, adverse drug reactions and have advantages in safety and tolerability as it is metabolized to EXP3174 with the aid of esterases in gastrointestinal tract. Furthermore, when compared with other traditional antihypertensive drugs, allisartan has advantage of potential cardiac and renal protective benefits [26, 27]. Allisartan was reported to be less toxic and highly effective in animal models [16]. However, till date no data is available on vascular remolding effect of allisartan. Therefore, the current study was aimed to evaluate the effects of allisartan in vascular remolding using MAs in hypertensive rats through voltage-gated potassium channels.

## Methods

### Experimental animals

A total of 47 Sprague Dawley (SD) rats were purchased from Shanghai Laboratory Animal Research Center (Shanghai, China) and were housed under standard conditions of temperature (22 ± 2 °C) and at 12 h of light–dark cycle. All experimental protocols were approved by the Institutional Animal Care and Use Committee of Shanghai Jiao Tong University (Shanghai, China) and the Ethics Committees of Shanghai Jiao Tong University. All experiments were performed in accordance to relevant guidelines and regulations in the Care and Use of Laboratory Animals. The study was conducted in accordance with the Basic & Clinical Pharmacology & Toxicology policy for experimental and clinical studies [28].

### Renal artery stenosis

Renal artery stenosis was performed according to the method described by Goldblatt et al. [29]. Briefly, left kidney of SD rats was exposed by left paracostal celiotomy after anesthetizing the animals with isoflurane inhalation. Blunt tipped vascular scissors and hooks were used to isolate renal artery, vein and nerve while left renal artery was clipped using a vascular clip and secured with nylon suture. A change in kidney color from dark brown to yellowish red was observed due to application of clip on the renal artery. Once the artery was clipped, the kidney was placed back in its original position and then the cavity was sutured in two layers (muscle and skin). During the surgery the body temperature was maintained by placing the animal in supine position on thermo controlled (37 °C) heating pad and monitored using digital rectal thermometer.

### Experimental procedure

Animals was randomly divided into two groups: sham operation control group (*n* = 14) and hypertension operation group (*n* = 33). Post renal artery stenosis, the hypertension operation group animals were randomly divided into two groups: allisartan treatment group (*n* = 15) and hypertension control group (*n* = 18). The animals in allisartan treatment group were administered with 10 mg/kg body weight allisartan (ShenZhen Salubris Pharmaceuticals Co. Ltd., Jinan, China) once a day through an oral gavage for 4 weeks.

### Preparation for wire myography of MAs

Rats were anesthetized with ketamine/xylazine or Sodium phenobarbital and sacrificed by exsanguination. Segment of the third-order MA were isolated from individual rats. The MA was removed, cleaned and segments (∼2 mm) were mounted in a myograph (Danish MyoTechnology, Aarhus, Denmark) for isometric tension recording. The composition of physiological salt solution (PSS) in the chamber was 125 mM NaCl, 4.6 mM KCl, 2.5 mMCaCl_2_, 25.4 mM NaHCO_3_, 1 mM Na_2_HPO_4_, 0.6 mM MgSO_4_ and 10 mM glucose, maintained at 37° C and aerated with 95 % O_2_ and 5 % CO_2_. MAs were equilibrated for 60 min before undergoing a passive force normalization procedure. The arterial segments were contracted with the α-1-adrenoceptor agonist methoxamine (10 µM in the MA), which was determined previously to produce a sub-maximal contraction (80–90 % of the maximal contraction) in the respective vessels. Flowing contraction with Norepinephrine, ML213 was applied in a manner of increasing concentration. The relaxation of MAs was measured by applying different doses (0.00001, 0.0001 and 0.001) of ML213. The response curves were constructed with results obtained. The MAs were depolarized using 60 mM KCl and then washed with PSS twice. For pressure myography, arteries were challenged with 60 mM KCl to test vessel viability. The artery was acclimatized for 20 min at 60 mmHg. Myogenic tone development was assessed over the intraluminal pressure range of 0–100 mmHg with pressure increments at 10 mm Hg every 3 min. The pressure curve was constructed by measuring outer-diameters at incremental steps of 10 mmHg from 0 to 100 mmHg (each step 3 min).

###  Real time polymerase chain reaction (RT-PCR)

Real time polymerase chain reaction **(**RT-PCR) was performed as described previously by Hedegaard et al., [30]. Briefly, using TissueLyser (Qiagen), the tissue samples were homogenized for 3 min and centrifuged for 2 min. Using TRIzol Reagent (Invitrogen, USA) and Qiacube (Qiagen), the total RNA was extracted and isolated, respectively, as per instructions of manufacturer. cDNA was synthesized using SuperScript™ III Reverse Transcriptase, SuperAse In and random decamer primers. GAPDH was used as house-keeping gene and relative quantification to estimate mRNA expression was performed. qRT–PCR was performed with the 2×SYBR green master mix (Takara, Japan) with a 7500 Real-Time PCR System (Applied Biosystems). The PCR primer sequences are listed below. All fold changes were calculated by the method of 2^−ΔΔCt^ in tissue sample.

- Rno-KV7.1_F CCATCTTTGTTCATCCCCATCT.
- Rno-KV7.1_R CCAGTTGTGTCACCTTGTCTT.
- Rno-KV7.2_F GGTGTCTCATTCTTCGCTCTT.
- Rno-KV7.2_R TCCGCCGTTTCTCAAAGTG.
- Rno-KV7.3_F ATACACATTTATCTGCTCTTCCTTTTA.
- Rno-KV7.3_R TGCTCTCAGTTTATCCGAATCAA.
- Rno-KV7.4_F GCTCATCTTCGCCTCTTTCC.
- Rno-KV7.4_R GCCAATGGTCGTCAGTGTAAT.
- Rno-KV7.5_F CCTGGCGTACACGAGAGTAT.
- Rno-KV7.5_R TTTGACTGGGCGAACTGAAC.
- Rno-GAPDH-F AGCTTCCCATTCTCAGCCTTGACT.
- Rno-GAPDH-R ACAAGATGGTGAAGGTCGGTGTGA.

### Statistical analysis

Experimental data were expressed as means ± SD when the study was performed at least three times. Differences were evaluated for statistical significance (*p* < 0.05) by ANOVA or t test with GraphPad Prism 8.0 (GraphPad Software, San Diego, CA, USA). Non-parametric test was applied for not normally distributed data and Kruskal Wallis test and Mann Whitney test was used to calculate statistical significance (*p* < 0.05).

## Results

### Effect of allisartan treatment on blood pressure

**Fig. 1:**
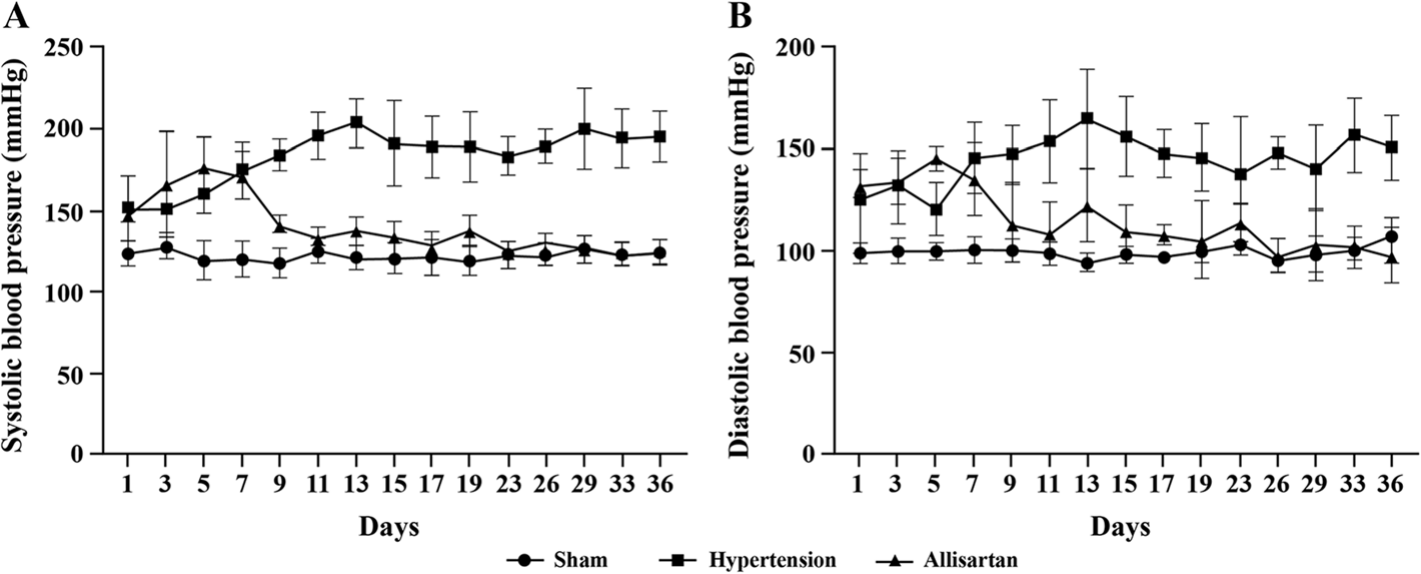
Effect of allisartan on blood pressure on different days. **A** Systolic blood pressure; **B** Diastolic blood pressure

**Fig. 2:**
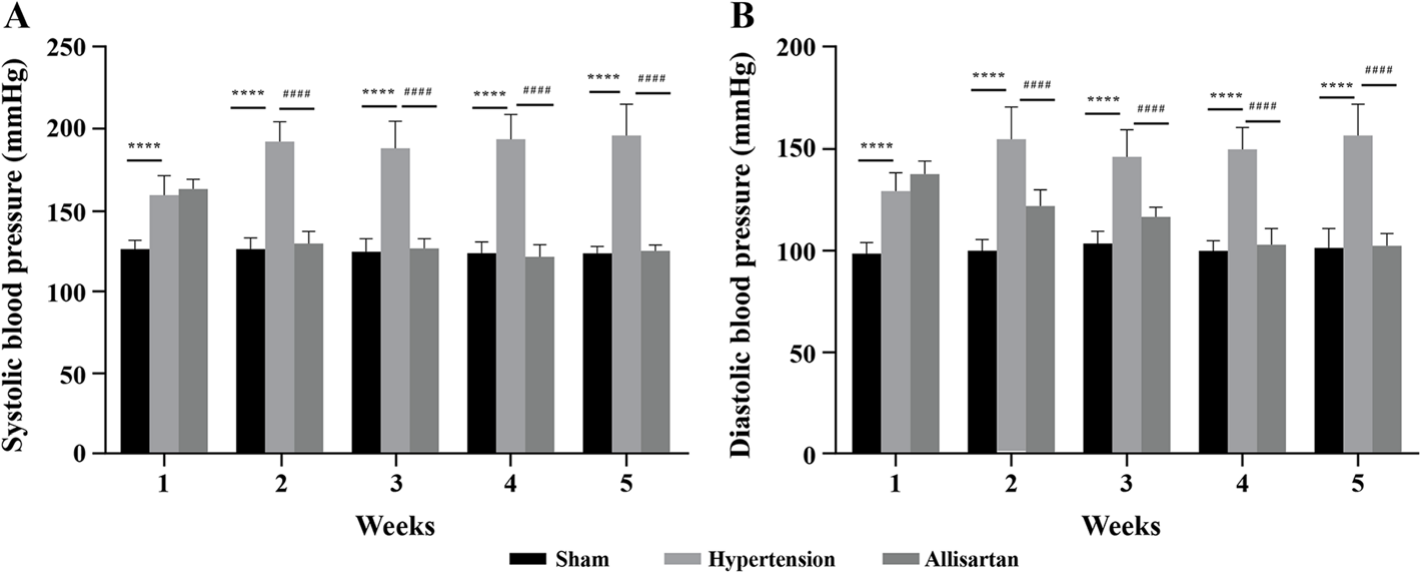
Effect of allisartan on blood pressure during different weeks. **A** Systolic blood pressure; **B** Diastolic blood pressure

After treatment with allisartan, the systoloc blood pressure (SBP) and diastolic blood pressure (DBP) were measured by the tail cuff method. A significant increase in mean arterial, SBP and DBP was observed in hypertension group compared to sham group (*P* < 0.05) while a significant decrease in mean arterial, SBP and DBP was observed in allisartan treatment group compared to hypertension control group (*P* < 0.05). Two weeks later, SBP and DBP were stable, and the SBP and DBP of animals in all 3 groups was observed two times every week. The results were represented in Fig. 1. Post renal artery stenosis, SBP was stable and reached to 170 mmHg during the first week. By end of 4th week the SBP and DBP of allisartan treatment group was comparable to that of sham control indicating effectiveness of allisartan (Fig. 2). A statistically significant difference was observed on comparing SBP of hypertension group Vs sham operation group (*P* < 0.0001) and allisartan treatment group Vs hypertension group (*P* < 0.0001) during all 4 weeks except for first week for allisartan treatment group Vs hypertension group (*P* = 0.8016).

### Morphological changes in aortic vessels

**Fig. 3:**
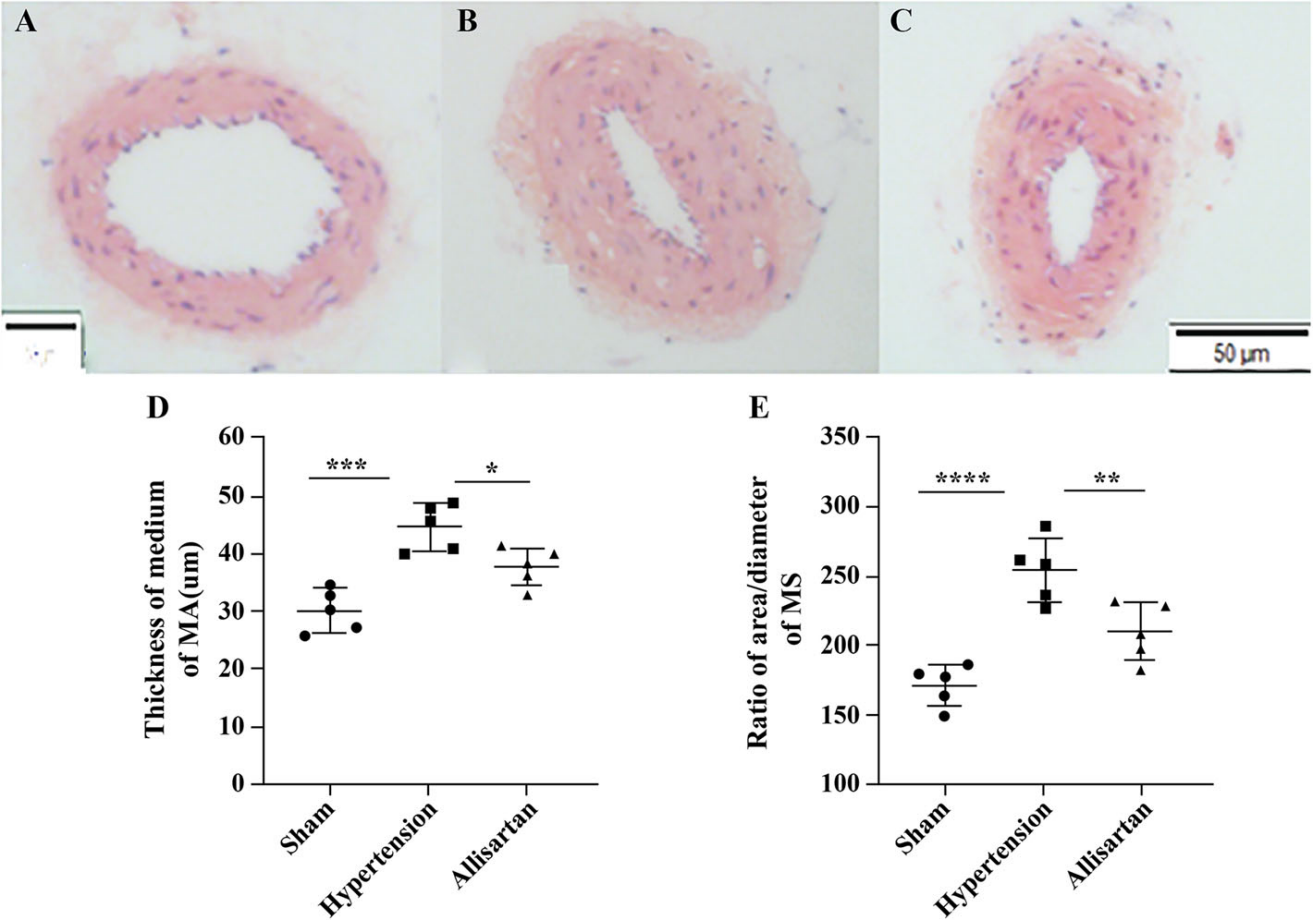
Effect of allisartan treatment on mesenteric arteries. **A** Control; **B** Hypertension; **C** Allisartan; **D** Thickness of mesenteric arteries; **E** Ratio of area and diameter

**Fig. 4:**
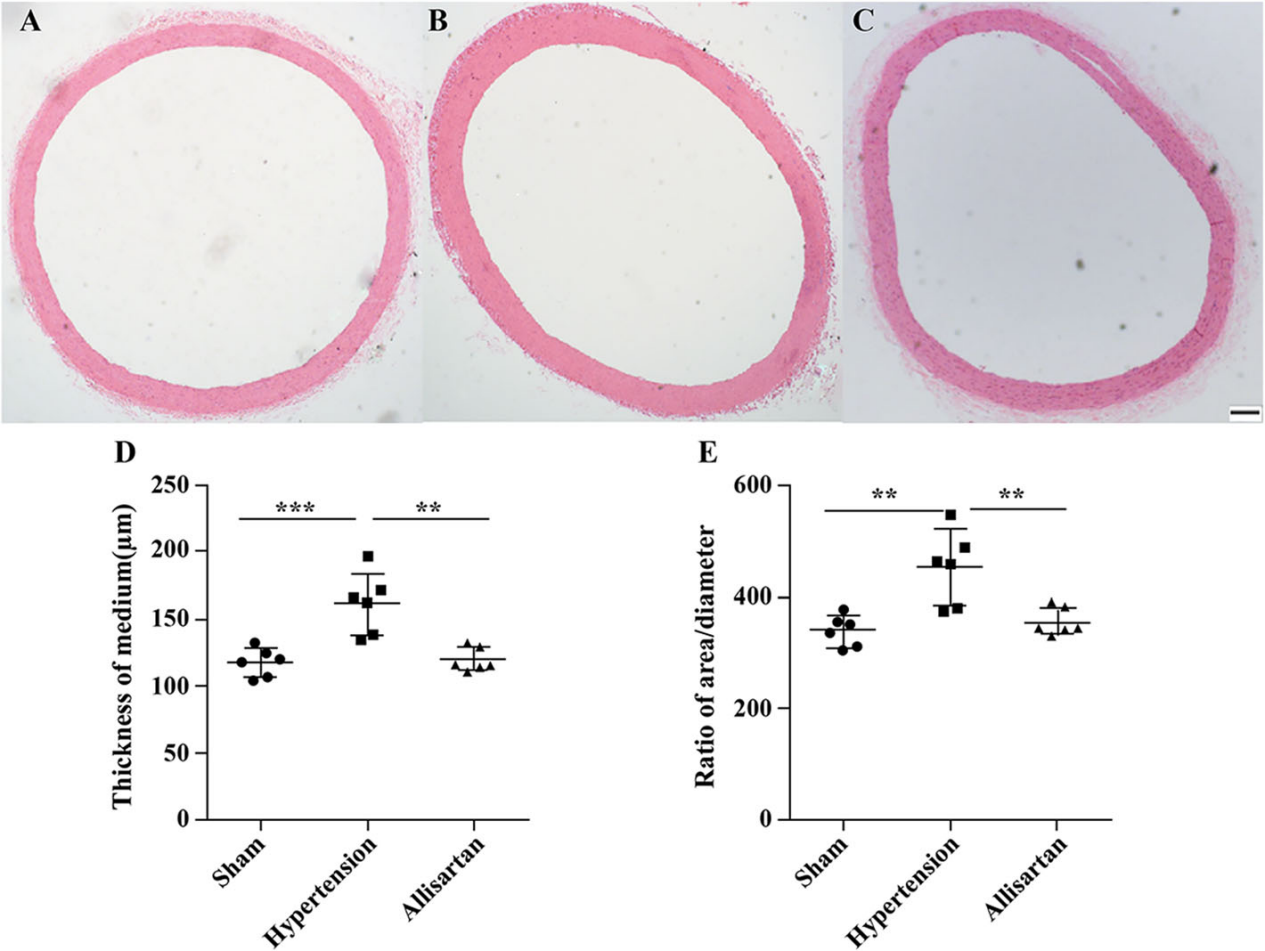
Effect of allisartan treatment on aorta. **A** Control; **B** Hypertension; **C** Allisartan; **D** Thickness of aorta; **E** Ratio of area and diameter

Post treatment with allisartan, the median thickness of aortic vessels was found to be 117.49 ± 10.38, 161.19 ± 20.55 and 120.33 ± 9.82 μm in the sham group, hypertension group and allisartan treatment group, respectively. A significant increase in median thickness of MAs and aorta was observed in hypertension group compared to sham group (*P* < 0.05) while a significant decrease was observed in allisartan treatment group compared to hypertension group (*P* < 0.05). The ratio of area to diameter of aorta was found to be 335, 451.72 and 357.26 in sham group, hypertension group and allisartan treatment group, respectively. Similar to median thickness, the ratio of area to diameter of both MAs and aorta also showed a significant increase in hypertension group compared to sham group (*P* < 0.05) while a significant decrease was observed in allisartan treatment group compared to hypertension group (*P* < 0.05; Figs. 3 and 4).

### Electrophysiological changes

**Fig. 5:**
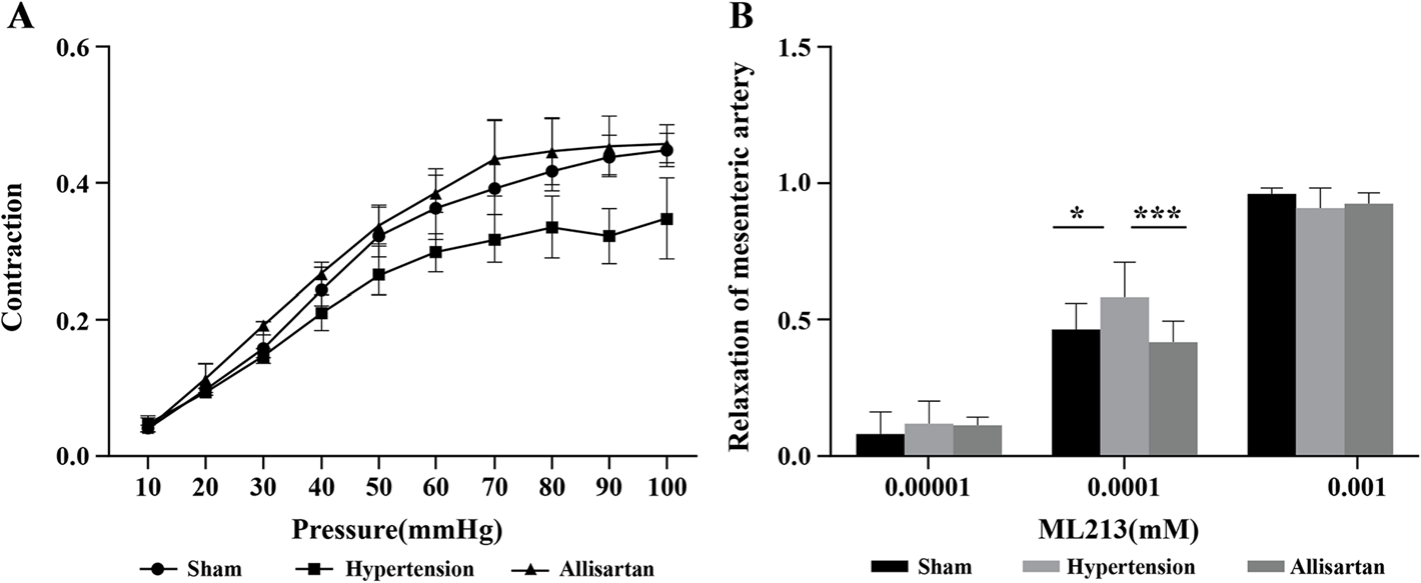
Wire myography of mesenteric arteries. **A** Using varying pressures; **B** Using ML213

Dose response curves of carbochol obtained from wire myography demonstrated higher contraction of MAs in the allisartan treatment and sham groups compared to MAs of hypertension group and the contraction was found to increase with increase in dose of carbochol. After repolarizing the arteries, an increase in contraction of MAs with increase in pressure in all 3 groups was observed. The contraction of MAs of sham group and allisartan treatment group was higher compared to MAs isolated from hypertension group. The contraction of MAs isolated from allisartan treated group was comparable to that of sham group. With increase in concentration of ML213 from 0.00001 mM to 0.001 mM, the relaxation of mesenteric artery also increased. A significant increase in relaxation of mesenteric artery was observed in hypertension group compared to sham group (*P* < 0.05) whereas a significant decrease in allisartan treatment group compared to hypertension group (*P* < 0.05) at 0.0001 mM of ML213 (Fig. 5).

### mRNA expression in mesenteric artery and aorta

**Fig. 6:**
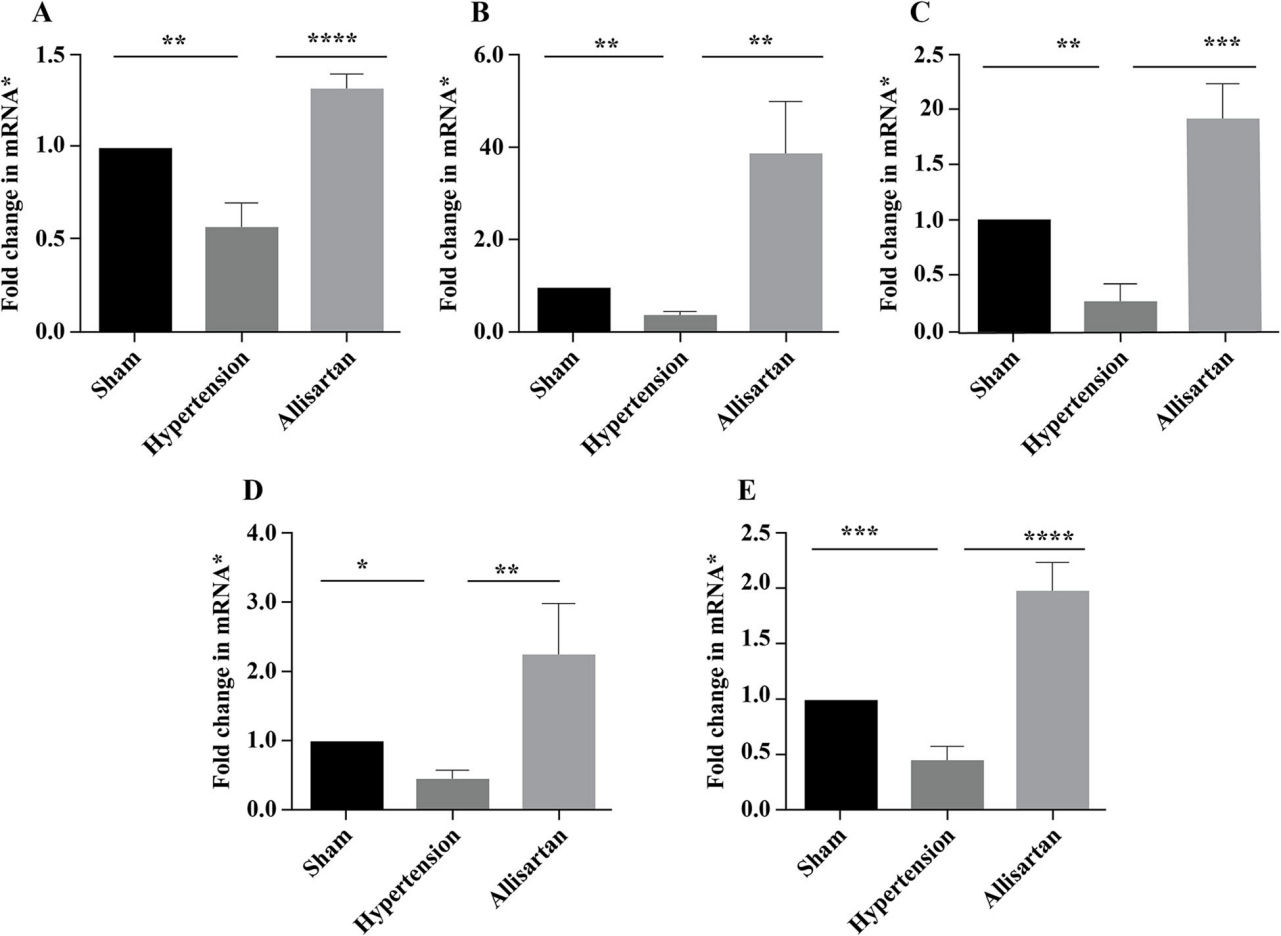
Expression of different potassium channel (Kv) subunits in mesenteric artery. **A** Kv7.1; **B** Kv7.2; **C** Kv7.3; **D** Kv7.4; **E** Kv7.5. *GADPH was used as house-keeping gene

**Fig. 7:**
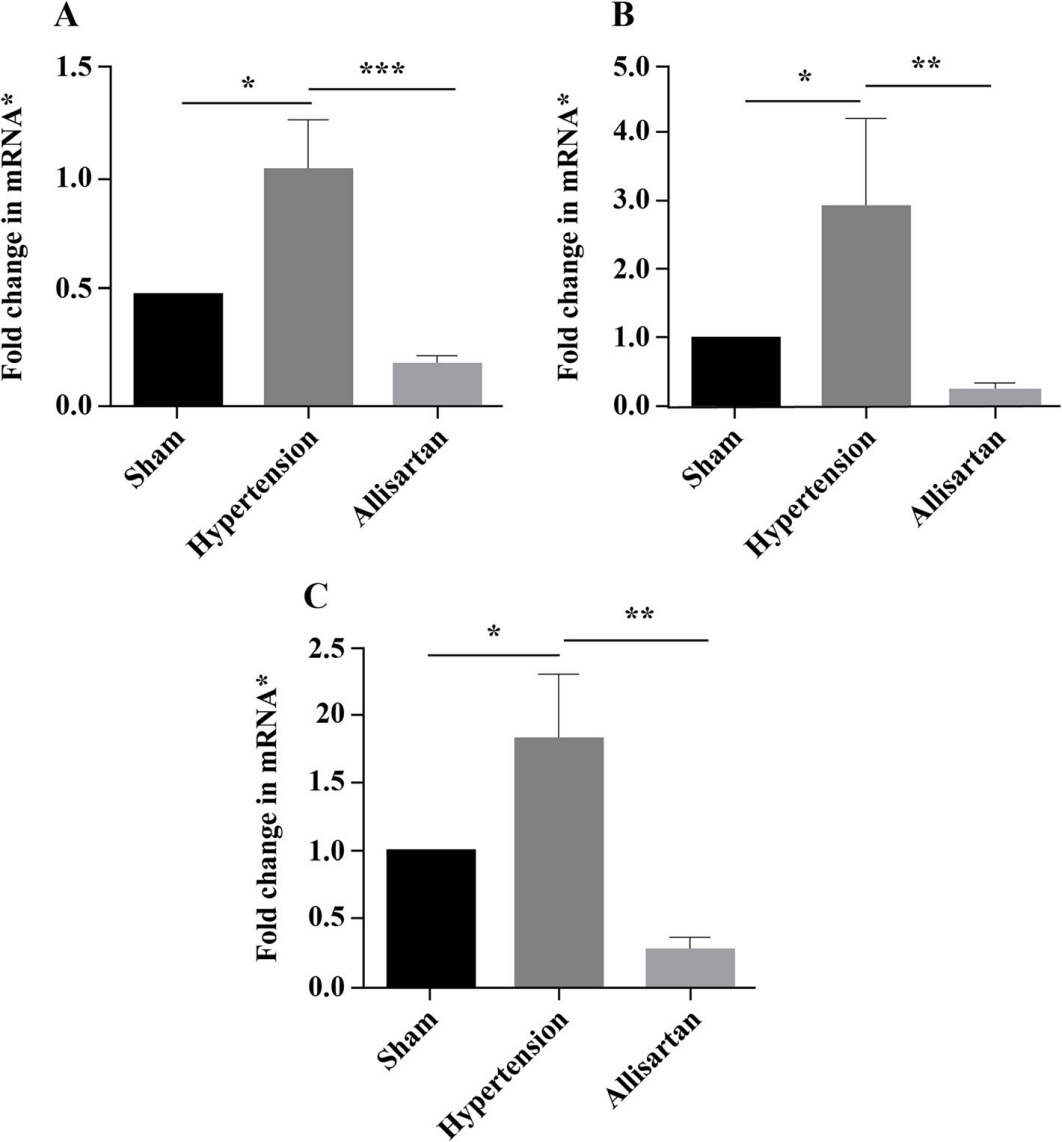
Expression of different potassium channel (Kv) subunits in aorta. **A** Kv7.1; **B** Kv7.2; **C**. Kv7.4. *GADPH was used as house-keeping gene

The expression of Kv7.1, Kv7.2, Kv7.3, Kv7.4 and Kv7.5 mRNA in the hypertension group was down-regulated significantly compared with the sham group (*P* < 0.05) while significantly up-regulated in allisartan treatment group compared with the hypertension group (*P* < 0.05) In MA, (Fig. 6). The expression of Kv7.1, Kv7.2 and Kv7.4 mRNA were significantly up-regulated in hypertension group compared with the sham group (*P* < 0.05) and were significantly down-regulated in allisartan treatment group compared with the hypertension group (*P* < 0.05) in aorta (Fig. 7). No significant difference was observed in Kv7.3 and 7.5 mRNA expression in aorta in all three groups.

## Discussion

Hypertension is associated with structural changes such as reduction in lumen diameter, increase in M/L ratio and resistance in the blood vessels which is considered as “vascular remodeling” [31]. ARBs are widely used in treating hypertension and related comorbidities. Losartan is a well-established ARB while allisartan is a newly developed ARB in China. Both of the drugs are metabolized to the same active metabolite, EXP3174 which is responsible for therapeutic effect. Unlike losartan, allisartan presents with an advantage of reduced metabolites making it less toxic compared to losartan [15]. Studies have reported role of allisartan in lowering BP and protective effects on heart, kidney and prevents vascular damage. But the mechanism of lowering BP and vascular remodeling through Kv is not established [15, 32]. The findings of the present study bridged this gap by demonstrating the vascular remodeling effects of allisartan through voltage-gated potassium channels in hypertensive rats. The results demonstrated reduced BP, reduced median wall thickness of MA and ratio of area/diameter of MA. The mRNA expression of Kv7.1, Kv7.2, Kv7.3, Kv7.4 and Kv7.5 mRNA subunits was observed to be up-regulated in MAs while Kv7.1, Kv7.2 and 7.4 mRNA significantly down-regulated in aorta of animals treated with allisartan compared to hypertension induced untreated animals.

In a study by Wu et al., comparing allisartan and losartan in hypertensive rats, reported less toxicity with allisartan compared to losartan. The study also reported long-lasting effects of allisartan on lowering SBP [16]. In par with the above study, the present study also showed reduced BP with allisartan treatment compared to untreated hypertension group. The reduction in SBP and DBP was observed from second week with allisartan treatment which was in accordance with previously published studies [32]. In the present study also median wall thickness and area/diameter ratio of MA was found to reduce with allisartan indicating benefit of allisartan in vascular remodeling.

ML213 is a novel Kv7 channel activator. It is reported to have vasorelaxant effect in various blood vessels. ML213 was reported to have concentration-dependent relaxation effects [33]. In the present study, an increase in relaxation of MA was observed with increasing ML213 concentration. Thus, substantiated the involvement of Kv7 channels in ameliorating vascular remodeling. Inhibition of Kv channels decreases the outflow of K^+^ and increases the inflow of Ca^2+^ resulting in membrane depolarization and vasoconstriction. It also inhibits the cell apoptosis cycle, which further causes the contraction, increase and proliferation of smooth muscle cells, and promotes the thickening of smooth muscle layer [34–36]. Of these, Kv7 family was reported to regulate myogenic and vasoconstrictor-induced tone in different blood vessels [36]. So, in the present study the expression of different Kv7 mRNA channel subunits in MA and aorta were studied. The results showed up-regulation of all subunits of Kv7 channel in allisartan treatment group compared to untreated hypertension group indicating ameliorating effect of allisartan on vascular remodeling through Kv7 channels.

To the best of our knowledge, this is the first study to analyze the role of Kv7 subunits in ameliorating effect of allisartan on vascular remodeling in hypertensive animals. The present study is not without limitations, the animals were induced hypertension by renal artery stenosis which does not completely resemble hypertension in humans. The study lacks direct measurement of outward potassium current.

## Conclusions

We observed an up-regulation of all subunits of Kv7 channel in allisartan treatment group compared to untreated hypertension group. From the results, allisartan was found to lower BP and preserve vascular remodeling through Kv7 channels.

## Data Availability

The datasets used and/or analyzed during the current study are available from the corresponding author on reasonable request.

## Abbreviations

ARBs: Angiotensin II receptor blockers
CE: Angiotensin-converting enzyme
CVD: Cardiovascular disease
CYP450: Cytochrome P450
DBP: Diastolic blood pressure
Kv7: Voltage gated potassium channels
MA: Mesenteric arteries
PSS: Physiological salt solution
RAS: Renin-angiotensin system
RT-PCR: Real time polymerase chain reaction
SBP: Systolic blood pressure
SD: Sprague Dawley
